# Psychological flexibility and its correlations with and predictive utility for emotional wellbeing, fatigue, insomnia, and post-traumatic growth in cancer patients undergoing treatment

**DOI:** 10.3389/fpsyg.2026.1768998

**Published:** 2026-03-12

**Authors:** Francisco García-Torres, Ángel Gómez-Solís, Rosario Castillo-Mayén, Raquel Espejo-Siles, Francisco Jurado-González, Marcin Jablonski, Daniel Galera Bernabé, María José Jaén-Moreno, Enrique Aranda

**Affiliations:** 1 Department of Psychology, University of Cordoba, Córdoba, Spain; 2 Maimonides Biomedical Research Institute of Cordoba (IMIBIC), Córdoba, Spain; 3 Reina Sofía University Hospital of Cordoba, Córdoba, Spain; 4 Jan Kochanowski University in Kielce, Kielce, Poland; 5 Psychosomatic Research Center, Oral Oncology Center, School of Dentistry, São Paulo State University (UNESP), Araçatuba, São Paulo, Brazil; 6 Department of Social Health Sciences, Radiology and Physical Medicine, University of Cordoba, Córdoba, Spain; 7 Medical Oncology Department, Reina Sofía University Hospital, Córdoba, Spain

**Keywords:** cancer, oncology, psychological flexibility, fatigue, insomnia, post-traumatic-growth

## Abstract

**Objective:**

The use of acceptance and commitment therapy has shown favorable results in cancer patients. However, the role of psychological flexibility has yet to be determined.

**Methods:**

Patients with breast, colorectal, lung and gynaecological cancers in active treatment in stages I-III were invited to complete questionnaires assessing psychological flexibility (AAQ-II), anxiety and depression (HADS), fatigue (BFI), insomnia (ISI) and post-traumatic growth (PTGI-SF). Correlation and regression analyses were performed controlling for possible confounding variables.

**Results:**

One-hundred and fifty patients completed the questionnaires. The majority were married women with stage III breast cancer, primary-level education and a mean age of 53. On average, 18 months had passed since diagnosis, and surgery, chemotherapy and radiotherapy were the most frequent treatments. Results showed psychological flexibility correlated with and had predictive ability for fatigue, anxiety, depression and insomnia, but not for post-traumatic growth. Anxiety was significantly related to depression, fatigue and insomnia, and depression to fatigue, insomnia and post-traumatic growth. Fatigue was related to insomnia.

**Conclusion:**

Psychological flexibility appears to be a relevant variable to be taken into account in the treatment of cancer patients.

## Introduction

Cancer continues to be a relevant health issue worldwide. It is estimated that around 18.7 million new cases were diagnosed worldwide in 2022, and this incidence is expected to reach 32.6 million by 2050, with lung, breast and colorectal being the most common cancer types (International Agency for Research on Cancer, 2025). In Spain, a similar pattern can be observed, with 296,103 new cases expected to be diagnosed in 2025, reaching 350,000 by 2050. Similarly, colorectal, breast and lung cancer are the types with the highest incidence (Sociedad Española de Oncología Médica, n.d.).

In cancer patients, treatment-associated symptoms such as fatigue are common. This fatigue is experienced as a state of overwhelming exhaustion and a reduced capacity to perform physical and mental tasks that is not relieved by rest, resulting in a high level of discomfort (Al Maqbali et al., 2021). Cancer-related fatigue can appear in up to 62% of patients undergoing treatment. Predictor factors are female sex, younger age, and cancer as well as treatment type (DSilva et al., 2023). Sleep disturbances have been associated with fatigue in cancer patients. In this population, symptoms manifest themselves in different ways. They affect the onset, maintenance and depth of sleep, and cause early awakening, thereby affecting the satisfaction gained from sleep (Gellman, 2020). Recent data suggest that 57.4% of cancer patients may experience sleep problems, while being female, having a lower level of education, and undergoing certain types of treatment, chemotherapy and radiotherapy in particular, are predictors for higher levels of insomnia or poor sleep quality (Chen et al., 2024; Zhao et al., 2025).

Generally, an anxiety response can be an adaptive tool in a threatening situation such as a cancer diagnosis. When this response is too prolonged and severe, however, and affects all spheres of the patient’s life, it becomes a clinical problem. This is the case for up to 28% of patients (Grassi et al., 2023). In addition to anxiety, depression is also frequent, affecting one in four patients, as cancer diagnoses and treatments often trigger feelings of sadness and loss. If such feelings persist and become more severe, it can lead to depression. Previous studies have identified various factors that are associated with the occurrence of anxiety and depression in patients, such as being female, of a younger age, of a low socio-economic level, and undergoing chemotherapy treatment. Other relevant factors are the type of cancer (particularly pancreatic, lung or thyroid cancer), the duration of the disease, and being unemployed (Grassi et al., 2023). Conversely, a traumatic event such as a cancer diagnosis can also cause other profound changes in patients’ lives. For some, the threatening experience of the disease is experienced as an opportunity for change, promoting a search for deeper meaning, which is known in literature as post-traumatic growth (Ata et al., 2023). In this context, research shows that age, level of education and time elapsed since diagnosis correlate with post-traumatic growth in such patients (Boyacıoğlu et al., 2022; Yeon and Yun, 2022).

Psychological flexibility (PF), a core concept of Acceptance and Commitment Therapy (ACT), can be defined as the ability to be fully aware of the present moment and committed to one’s core personal values (Hayes et al., 2006). According to the ACT model, PF is founded on six key processes: acceptance, cognitive defusion, contact with the present moment, the self as context, clarifying values, and committed action (Hayes et al., 2006). In cancer patients, ACT has shown positive results in improving anxiety and depression, but the effect of this therapy on PF and other relevant symptoms such as fatigue and insomnia remains inconclusive, probably owing to the limitations of the research designs used and the heterogeneity of interventions (García et al., 2023; Sauer et al., 2024).

As regards PF, there are few studies that have examined possible correlations with relevant aspects in cancer patients. Previous research has observed relations between PF and emotional distress in prostate and thyroid cancer patients (McAteer and Gillanders, 2019; Sevier-Guy et al., 2021), and with fatigue in breast cancer patients (Novakov, 2011). Only one study has observed relationships between PF and post-traumatic growth in patients who underwent radiotherapy for different types of cancer (Akcan et al., 2024). With regard to insomnia, no studies have been found to date that have assessed its relationship with PF. It is necessary, therefore, to provide further evidence to be able to fully determine the possible correlations and the predictive role that PF may have with regard to symptoms which negatively influence the wellbeing of cancer patients undergoing treatment. Hence, presuming that PF does play a relevant role in cancer patients, it would be useful to clearly define its correlations with and ascertain its predictive utility for anxiety, depression, fatigue, insomnia, as well as post-traumatic growth.

## Materials and methods

### Design, setting and participants

This retrospective study involved a total of 217 cancer patients undergoing treatment agreed to participate in this study, and 150 completed the questionnaires. Reasons for not participating were: inability to attend meeting(s) (*n* = 4), treatment side effects (*n* = 12), patient’s passing (*n* = 2), distance or work (*n* = 50). The inclusion criteria for participation in the study were the following: men or women, aged between 18 and 70, with a confirmed stage I-III cancer diagnosis for breast, colorectal, gynaecological or lung cancer, who were eligible for treatment, and not currently involved in any other study. Study participants were recruited at the Reina Sofía University Hospital in Córdoba (Spain). The researchers provided the nursing staff of the Day Hospital Oncology Unit with the inclusion and exclusion criteria for the study to pre-select potential candidates before inviting them to participate in the study. When a patient expressed interest in participating, a member of the research team provided them with a written informed consent form to be signed by the participant, which stated the objectives of the study, expressed guarantees with regard to data confidentiality, and explained that participants had the right to leave the study at any point without incurring any negative consequences.

### Instruments

#### Action and acceptance questionnaire II (AAQ-II)

This instrument was designed to assesses the opposite of psychological flexibility (psychological inflexibility) using 7 items that are assessed on a scale ranging from 1 (never true) to 7 (always true), resulting in overall scores ranging between 7 and 49, where higher values indicate greater psychological inflexibility. Scores above 25 can be interpreted as meaning a person’s psychological inflexibility has a negative impact on their wellbeing. The instrument has adequate psychometric properties in Spanish version. (*α* = 0.93) (Ruiz et al., 2013).

#### Hospital anxiety and depression scale (HADS)

This scale is widely used in oncology settings to assess anxiety and depression. It consists of 14 items divided into two subscales of 7 items each for anxiety (HADS-A) and for depression (HADS-D). The sum of the response scores ranges from 0 to 21, where a higher score indicates an increased occurrence of anxiety and depression symptoms. A score of ≥ 8 is considered an indicator of probable anxiety and depression (Grassi et al., 2023). The scale has demonstrated good psychometric properties in Spanish version in oncological setting (*α* = 0.80–0.87) (Terol-Cantero et al., 2015).

#### Brief fatigue inventory (BFI)

This instrument was designed to assess the severity and impact on daily functioning of fatigue in cancer patients. It opens with an initial question asking patients whether they have experienced greater fatigue than usual in the previous week, which is answered in the affirmative or negative. This is followed by 4 questions relating to fatigue experienced at the current time and in the period 24 h prior, which are answered on a scale from 0 (no fatigue) to 10 (worst fatigue imaginable). It also includes 6 questions regarding the interference of fatigue in the patient’s daily life, answered on a scale from 0 (no interference) to 10 (maximum interference). The sum of the scores indicates the degree of fatigue experienced; the higher the score, the greater the fatigue. Scores can be categorized as mild (International Agency for Research on Cancer, 2025; Sociedad Española de Oncología Médica, n.d.; Al Maqbali et al., 2021), moderate (DSilva et al., 2023; Gellman, 2020; Chen et al., 2024), and severe (Zhao et al., 2025; Grassi et al., 2023; Ata et al., 2023; Boyacıoğlu et al., 2022). This inventory has demonstrated good psychometric properties in a Spanish population of cancer patients (*α* = 0.96–0.97) (Lorca et al., 2016).

#### Insomnia severity index (ISI)

This instrument was designed to assess the severity of sleep problems by examining different sleep-related aspects: onset, maintenance, early awakening, as well as perceived satisfaction with sleep, interference of sleep problems with daily life, the degree to which others are aware of them, and the degree of distress and worry associated with them. It uses 7 items measured on a scale ranging from 0 (not at all) to 4 (very severe/very dissatisfied/very much). The sum of the scores ranges from 0–28. A higher score indicates a greater presence of sleep-related problems. The scores can be divided into the following categories: non-significant insomnia (0–7), subthreshold insomnia (Grassi et al., 2023; Ata et al., 2023; Boyacıoğlu et al., 2022; Yeon and Yun, 2022; Hayes et al., 2006; García et al., 2023; Sauer et al., 2024), moderate insomnia (McAteer and Gillanders, 2019; Sevier-Guy et al., 2021; Novakov, 2011; Akcan et al., 2024; Ruiz et al., 2013; Terol-Cantero et al., 2015; Lorca et al., 2016), and severe insomnia (Fernández-Mendoza et al., 2012; Castro et al., 2015; Fawson et al., 2024; Shin et al., 2024; Wang et al., 2025; Kopecky et al., 2026; Yanez et al., 2024). The psychometric properties of the instrument have proven to be adequate in a Spanish sample (*α* = 0.82) (Fernández-Mendoza et al., 2012).

#### Post-traumatic growth inventory short form (PTGI-SF)

This brief inventory assesses post-traumatic growth (positive changes that some people experience after suffering a traumatic event) through 10 items that are answered on a scale from 1 (completely disagree) to 6 (completely agree). The resulting scores of the different items are added up. A higher score is interpreted as greater post-traumatic growth. The instrument has adequate psychometric properties in its Spanish version (*α* = 0.83–0.93) (Castro et al., 2015).

Participants also completed a socio-demographic questionnaire designed to collect information relating to sex, age, time elapsed since diagnosis, education, employment, annual income, marital status, cancer type, treatment and stage.

### Statistical analysis

First, descriptive statistics (means, standard deviations, and frequencies) were obtained for the study variables. Subsequently, Pearson’s bivariate correlation analyses were conducted between the study variables: psychological flexibility (AAQ-II), fatigue (BFI), insomnia (ISI), post-traumatic growth (PTGI-SF), and anxiety and depression using the HADS subscales for each (HADSA and HADS, respectively). Finally, a hierarchical multiple regression analysis was performed to test the predictive utility of psychological flexibility for the dependent variables (fatigue, insomnia, anxiety, depression, and post-traumatic growth). In order to control for the possible effect of confounding variables, they were introduced in a first forced-entry block, and the predictor variable in the second block using a stepwise method. The categorical independent variables were coded as dummy variables to enter the values in the different models. All analyses were performed using IBM SPSS Statistics v.29 for Windows. The results are significant if *p* < 0.05.

### Ethical considerations

Before the study was carried out, the study received approval from the Andalusian Biomedical Research Ethics Portal (ref. 5,090).

## Results

The clinical and socio-demographic data of the study sample are shown in Table 1. Most participants were women, of a median age of 53, married, with primary-level education, and on sick leave due to cancer. The most frequently reported pathology was stage III breast cancer. A combination of surgery, chemotherapy, and radiotherapy was the most common form of treatment. The median time since diagnosis was 18 months.

**Table 1:** Clinical and sociodemographic data of the participants.

	Frequency (%)
Gender	
Female	142 (94.7)
Male	8 (5.3)
Marital status	
Single	14 (9.4)
Married	104 (69.8)
With partner	7 (4.7)
Separated	4 (2.7)
Divorced	16 (10.7)
Widow	2 (1.3)
Other	2 (1.3)
Education	
Primary	43 (28.9)
Vocational	38 (25.5)
Secondary	34 (22.8)
Advanced	34 (22.8)
Employment	
Full-time job	11 (7.4)
Part-time job	6 (4.1)
Seasional	1 (0.7)
Unemployed	18 (12.2)
Sick leave	71 (48)
Homemaker	12 (8.1)
Retirement Self-employment	25 (16.9) 4 (2.7)
Cancer type	
Breast	122 (81.3)
Colorectal	13 (8.7)
Gynecologic	10 (6.7)
Lung	5 (3.3)
Cancer stage	
I	18 (15)
II	40 (33.3)
III	61 (51.7)
Not answered/not Known	30 (20)
Treatment	
Chemotherapy	39 (26.4)
Radiotherapy	1 (0.7)
Surgery and chemotherapy	37 (25)
Surgery and radiotherapy	2 (1.4)
Chemotherapy and radiotherapy	5 (3.4)
Surgery, chemotherapy, and radiotherapy	64 (43.2)

	*M* (*SD*)
Age	53.18 (8.88)
Months after diagnosis	18.11 (27.26)

The correlation analyses performed are shown in Table 2. The results show that anxiety significantly correlates with depression, psychological flexibility, fatigue and insomnia. Depression correlates with psychological flexibility, fatigue with insomnia and post-traumatic growth (inverse), while psychological flexibility also correlates with fatigue and insomnia, and fatigue with insomnia.

**Table 2:** Correlation analysis between the study variables.

Variables	Mean (*SD*)	1	2	3	4	5	6
1. HADS-A	7.94 (4.37)	–	–	–	–	–	–
2. HADS-D	5.32 (3.80)	0.55***	–	–	–	–	–
3. AAQII	21.51 (9.94)	0.60***	0.57***	–	–	–	–
4. BFI	33.82 (22.68)	0.45***	0.59***	0.47***	–	–	–
5. ISI	10.56 (6.27)	0.49***	0.40***	0.40***	0.53***	–	–
6. PTGI-SF	45.31 (10.37)	−0.03	−0.26***	0.00	−0.003	0.05	–

***p < 0.001. HADS-A, Hospital Anxiety and Depression Scale, Anxiety; HADS-D, Hospital Anxiety and Depression Scale, Depression; BFI, Brief Fatigue Inventory; ISI, Insomnia severity Index; PTGI-SF, Post-traumatic growth inventory short form.

The results of the regression models showed that after controlling for possible confounding variables, the predictors explained 28% of the variance in fatigue [*R*2 = 0.28, *F*(14,133) = 3.79, *p* < 0.001], in particular sex (*β* = –20.43, *p* = 0.01) and PF (*β* = 1.00, *p* < 0.001). For insomnia, the model is also significant, explaining 21% of the variance [*R*2 = 0.21, *F*(10,137) = 3.77, *p* < 0.001], while only PF obtained significant scores (*β* = 0.25, *p* < 0.001).

For anxiety, the model is significant, explaining 45% of variance [*R*2 = 0.45, *F*(22,87) = 3.32, *p* < 0.001], with colorectal cancer (*β* = –4.43, *p* < 0.003) and PF (*β* = 0.24, *p* < 0.001) as significant predictors. The prediction model for depression is significant [*R*2 = 0.50, *F*(22,87) = 4.09, *p* < 0.001], explaining 50% of the variance. Lung cancer (*β* = 4.57, *p* = 0.01) and PF obtained significant scores (*β* = 0.24, *p* < 0.001). In conclusion, the different variables demonstrated no predictive ability for post-traumatic growth. The complete regression tables can be found in Supplementary materials.

## Discussion

In cancer patients undergoing treatment, the role of PF and its relationships and predictive utility for relevant symptoms such as anxiety, depression, fatigue, insomnia, and post-traumatic growth remains inconclusive (Sauer et al., 2024; Akcan et al., 2024; Fawson et al., 2024).

In the present study, the data obtained shows that in a large group of cancer patients greater psychological inflexibility is indeed positively related to higher levels of anxiety and depression, which is in line with other results previously observed in cancer patients (McAteer and Gillanders, 2019; Sevier-Guy et al., 2021). As regards anxiety, the regression model shows that colorectal cancer and PF act as significant predictors. These results may be due to the fact that a high percentage of participants in the sample had undergone chemotherapy, radiotherapy, and surgery, which is associated with higher anxiety according to previous literature on patients with colorectal cancer, particularly during the 2 first years after diagnosis (Shin et al., 2024; Wang et al., 2025; Kopecky et al., 2026).

As for depression, the model shows that low income, lung cancer and PF act as predictors. Previous studies had already pointed out that low income levels correlated with depression in this group of patients, probably because people with lower income levels have greater difficulties in obtaining or maintaining employment and accessing mental health services (Yanez et al., 2024). In addition, in lung cancer patients, depression frequently sets in after the first chemotherapy session, probably as a result of accumulated stress, physical discomfort, and a lower expected survival rate (Luo et al., 2024; Tian et al., 2021). Taking into account that PF can be defined as a full connection with the present moment that helps establish patterns of behavior aligned with one’s goals and values, the results suggest that this way of coping with reality may help cancer patients in treatment by favoring their adaptation, and may improve their emotional wellbeing (Fawson et al., 2024).

In terms of fatigue and PF, relationships are observed and, furthermore, sex and PF emerge as predictors in the proposed models. With regard to sex, most of the study sample was made up of women. Taking into account that, according to recent data, women are up to 69% more likely to suffer from fatigue, this could explain the results obtained (Du et al., 2025). According to the authors of that study, the reasons that explain this greater affectation of fatigue in women may be due to differences in drug absorption, immune responses, and the fact that even during the treatment of the disease they continue to be in charge of household tasks. On the other hand, PF also appeared as a predictor of higher levels of fatigue in this group of patients. Previous studies have observed inconsistencies after the application of ACT to reduce fatigue. Positive effects were only observed in patients with advanced cancer (Fang P. et al., 2023). However, the results obtained in the present study are in line with those previously obtained by Novakov (2011) in a sample of breast cancer patients, where the author observes PF’s correlation with and predictive ability for fatigue. It is possible that people with greater psychological flexibility are more willing or able to engage in activities and make decisions in accordance with their values and interests despite the limitations that treatment imposes, which may improve how they experience fatigue (Chen and Li, 2025).

The results also indicate that PF correlates with and has predictive ability for insomnia in the sample. In the general population, relationships between PF and sleep problems have been observed by various authors. However, the underlying mechanism remains unclear (El Rafihi-Ferreira et al., 2024). It is possible that PF reduces a person’s obsession with thoughts and sensations, resulting in better sleep, owing to the paradoxical effect of excessive attempts to control sleep (Shin et al., 2023). This may explain the results obtained.

Lastly, previous studies have shown relationships between PF and post-traumatic growth in samples of mostly metastatic patients. It is possible that these relationships are due to the fact that at this point in the disease there is a greater need for patients to reevaluate their lives and available options, which is easier if there is greater psychological flexibility (Akcan et al., 2024). However, the participants in the present study were limited to the early stages of the disease, which may explain this observed lack of relationships. Further research is needed in this regard to clearly establish the influence of PF on post-traumatic growth.

### Relevance for clinical practice

Psychological flexibility is a key concept that relates to issues to consider in cancer patients, such as anxiety, depression, fatigue, insomnia and post-traumatic growth but to date, the results are inconsistent. The results indicate that psychological flexibility is related to and predictive of the occurrence of anxiety, depression, fatigue, and insomnia in these patients, but not with posttraumatic growth. Early assessment of psychological flexibility could help prevent the onset of these symptoms in cancer patients undergoing treatment.

This study has limitations that should be taken into account when interpreting the results described. Firstly, the analyses were performed with data from a cross-sectional design, so it would be advisable to confirm these data using longitudinal designs. In addition, participants were mostly breast cancer patients, with a lower presence of patients affected by other types of cancer, which may limit the interpretation of the results due to the heterogeneity of the study sample. Future studies should include samples that take this variability into account and use samples with specific cancer types. Furthermore, most of the participants were women, suggesting that the results obtained in relation to sex should be treated with caution. In addition, although the instruments employed are common in cancer studies, the instrument used to assess psychological flexibility has generated controversy, as it assesses mostly discomfort and negative emotions using unclear language. Despite these limitations, other similar instruments such as the Brief Experiential Avoidance Questionnaire (BEAQ), or the Comprehensive Assessment of Acceptance and Commitment Therapy processes (CompACT) are not significantly better than the AAQ-II (Fang S. et al., 2023). Furthermore, the participation of a single center may affect the generalizability of the results obtained.

The results obtained in the present study point to the need to include PF in the evaluation of cancer patients on account of its observed relationships with frequently occurring symptoms such as anxiety, depression, fatigue and insomnia and invite us to explore the possible relationships between psychological flexibility and other relevant variables in cancer patients.

## Data Availability

The raw data supporting the conclusions of this article will be made available by the authors, without undue reservation.
