# Supplementary material for: Psychological flexibility and its correlations with and predictive utility for emotional wellbeing, fatigue, insomnia, and post-traumatic growth in cancer patients undergoing treatment
Source: Front Psychol. 2026 Mar 12;17:1768998. doi: 10.3389/fpsyg.2026.1768998 (PMC13017947; doi:10.3389/fpsyg.2026.1768998)
Supplement: Supplementary file 1 [file Supplementary_file_1.docx]

**Supplementary material.** *Regression analysis for Fatigue and Insomnia*

|  | *b* | SE *b* | *β* | *t* |  | *b* | SE *b* | *β* | *t* |
| --- | --- | --- | --- | --- | --- | --- | --- | --- | --- |
| **BFI**  Model 1 |  |  |  |  | **ISI**  Model 1 |  |  |  |  |
| **Constant** | 65.45 | 16.59 |  | 3.94*** |  | 8.33 | 2.57 |  | 3.24*** |
| **Sex** | -20.83 | 9.11 | -.21 | -2.28* |  | 1.72 | 2.39 | .06 | .71 |
| **Age** | -.19 | .22 | -.07 | -.84 |  |  |  |  |  |
| **Cancer type** |  |  |  |  |  |  |  |  |  |
| Lung | 8.22 | 10.60 | .06 | .77 |  |  |  |  |  |
| Colorectal | -11.04 | 7.25 | -.13 | -1.52 |  |  |  |  |  |
| Gynecologic | -2.94 | 7.94 | -.03 | -.37 |  |  |  |  |  |
| **Treatment** |  |  |  |  |  |  |  |  |  |
| Chemotherapy | -3.97 | 4.56 | -.07 | -.87 |  | -1.23 | 1.28 | -.08 | -.95 |
| Radiotherapy | -13.54 | 22.88 | -.04 | -.59 |  | -6.49 | 6.41 | -.08 | -1.01 |
| Surg+Chemo | -3.52 | 4.87 | -.06 | -.72 |  | -1.53 | 1.30 | -.10 | -1.18 |
| Surg+Radio | 4.20 | 16.49 | .02 | .25 |  | .94 | 4.55 | .01 | .20 |
| Chemo+Radio | 21.98 | 11.20 | .17 | 1.96 |  | 4.95 | 3.07 | .14 | 1.61 |
| **Education** |  |  |  |  |  |  |  |  |  |
| Vocational | 2.27 | 5.14 | .04 | .44 |  | .56 | 1.42 | .03 | .39 |
| Secondary | -.12 | 5.35 | -.002 | -.02 |  | 2.44 | 1.48 | .16 | 1.65 |
| Advanced | -.35 | 5.52 | -.007 | -.06 |  | 1.94 | 1.46 | .13 | 1.32 |
|  |  |  |  |  |  |  |  |  |  |
| Model 2  **Constant** | 33.99 | 15.89 |  | 2.14* | Model 2 | 3.38 | 2.55 |  | 1.32 |
| **Sex** | -20.43 | 8.19 | -.20 | -2.49* |  | 1.03 | 2.20 | .03 | .46 |
| **Age** | -.04 | .20 | -.01 | -.19 |  |  |  |  |  |
| **Cancer Type** |  |  |  |  |  |  |  |  |  |
| Lung | 8.24 | 9.53 | .06 | .86 |  |  |  |  |  |
| Colorectal | -5.22 | 6.59 | -.06 | -.79 |  |  |  |  |  |
| Gynecologic | -3.02 | 7.14 | -.03 | -.42 |  |  |  |  |  |
| **Treatment** |  |  |  |  |  |  |  |  |  |
| Chemotherapy | -1.55 | 4.12 | -.03 | -.37 |  | -.57 | 1.18 | -.04 | -.48 |
| Radiotherapy | -4.93 | 20.62 | -.01 | -.23 |  | -4.71 | 5.90 | -.06 | -.79 |
| Surg+Chemo | -3.11 | 4.38 | -.06 | -.71 |  | -1.28 | 1.19 | -.08 | -1.07 |
| Surg+Radio | -.43 | 14.84 | -.002 | -.02 |  | .52 | 4.18 | .01 | .12 |
| Chemo+Radio | 14.06 | 10.16 | .11 | 1.38 |  | 3.17 | 2.84 | .09 | 1.11 |
| **Education** |  |  |  |  |  |  |  |  |  |
| Vocational | 1.22 | 4.62 | .02 | .26 |  | .17 | 1.31 | .01 | .13 |
| Secondary | .02 | 4.80 | .00 | .005 |  | 2.23 | 1.36 | .15 | 1.64 |
| Advanced | 2.20 | 4.98 | .04 | .44 |  | 2.30 | 1.34 | .15 | 1.71 |
| **AAQ-II** | 1.002 | .17 | .44 | 5.37*** |  | .25 | .04 | .39 | 5.15*** |

Note. BFI (Brief Fatigue Inventory), ISI (Insomnia Severity Index) AAQII (Action and Acceptance Questionnaire- II) Fatigue: *R^2^* = .10 for Step 1; ∆R^2^ = .17 for Step 2 (*p* < .001). Insomnia: *R^2^* = .06 for Step 1; ∆R^2^ = .15 for Step 2 (*p* < .001). * *p* < .05, ** *p* < .01, *** *p* < .001.

**Supplementary material 1**. *Regression analysis for Anxiety and Depression*

|  | *b* | SE *b* | *β* | *t* |  | *b* | SE *b* | *β* | *t* |
| --- | --- | --- | --- | --- | --- | --- | --- | --- | --- |
| **HADSA**  Model 1 |  |  |  |  | **HADSD**  Model 1 |  |  |  |  |
| **Constant** | 17.01 | 4.15 |  | 4.09*** |  | .77 | 3.76 |  | .20 |
| **Sex** | .1.67 | 2.30 | -.08 | -.72 |  | .68 | 2.08 | .04 | .32 |
| **Age** | -.07 | .05 | -.16 | -1.40 |  | .05 | .05 | .13 | 1.16 |
| **Income** |  |  |  |  |  |  |  |  |  |
| <12.450 | .35 | 1.13 | .03 | .31 |  | 1.93 | 1.02 | .22 | 1.88 |
| 20.200-35.200 | -1.46 | 1.20 | -.13 | -1.21 |  | -.02 | 1.09 | -.003 | -.02 |
| 35.200-60.000 | -.93 | 1.62 | -.06 | -.57 |  | .77 | 1.47 | .05 | .52 |
| >60.000 | -3.47 | 2.68 | -.13 | -1.29 |  | -1.11 | 2.42 | -.04 | -.46 |
| **Treatment** |  |  |  |  |  |  |  |  |  |
| Chemotherapy | -1.09 | 1.08 | -.11 | -1.01 |  | .15 | .98 | .01 | .15 |
| Surg+Chemo | -.21 | 1.15 | -.02 | -.18 |  | -.20 | 1.04 | -.02 | .20 |
| Surg+Radio | -.08 | 4.78 | -.002 | -.01 |  | -.96 | 4.32 | -.02 | -.22 |
| Chemo+Radio | 4.27 | 2.67 | .18 | 1.60 |  | 4.00 | 2.41 | .19 | 1.65 |
| **Months from diagnosis** | .01 | .01 | .06 | .60 |  | .008 | .01 | .05 | .48 |
| **Employment** |  |  |  |  |  |  |  |  |  |
| Part-Time | -2.30 | 2.69 | -.10 | -.85 |  | .38 | 2.43 | .01 | .15 |
| Seasional | -.15 | 4.73 | -.003 | -.03 |  | -2.23 | 4.28 | -.05 | -.52 |
| Unemployed | -1.02 | 2.07 | -.08 | -.49 |  | 1.62 | 1.87 | .13 | .86 |
| Self-employed | -4.29 | 3.85 | -.13 | -1.11 |  | 1.39 | 3.48 | .04 | .39 |
| Sick leave | -2.76 | 1.76 | -.32 | -1.56 |  | .39 | 1.59 | .05 | .24 |
| Homemaker | -2.80 | 2.26 | -.17 | -1.23 |  | .23 | 2.05 | .01 | .11 |
| Retirement | -3.96 | 2.09 | -.34 | -1.89 |  | -1.06 | .189 | -.10 | -.56 |
| **Cancer type** |  |  |  |  |  |  |  |  |  |
| Lung | 1.34 | 2.55 | .05 | .52 |  | 5.07 | 2.30 | .24 | 2.20* |
| Colorectal | -4.30 | 1.71 | -.30 | -3.50* |  | -1.44 | 1.55 | -.11 | -.93 |
| Gynecologic | -1.35 | 1.79 | -.08 | -.75 |  | -1.89 | 1.61 | -.12 | -1.17 |
|  |  |  |  |  |  |  |  |  |  |
| Model 2  **Constant** | 13.31 | 3.55 |  | 3.74*** | Model 2 | -2.29 | 3.06 |  | -.95 |
| **Sex** | .3.55 | 1.96 | -.18 | -1.80 |  | -1.20 | 1.69 | -.07 | -.70 |
| **Age** | -.08 | .04 | -.16 | -1.76 |  | .05 | .04 | .12 | 1.34 |
| **Income** |  |  |  |  |  |  |  |  |  |
| <12.450 | .38 | .96 | .04 | .40 |  | 1.97 | .82 | .23 | 2.38* |
| 20.200-35.200 | -.77 | 1.02 | -.07 | -.75 |  | .66 | .88 | .06 | .75 |
| 35.200-60.000 | .09 | 1.38 | .006 | .06 |  | 1.80 | 1.18 | .13 | 1.51 |
| >60.000 | -1.81 | 2.27 | -.06 | -.79 |  | .54 | 1.95 | .02 | .27 |
| **Treatment** |  |  |  |  |  |  |  |  |  |
| Chemotherapy | -1.33 | .91 | -.13 | -1.45 |  | -.08 | .78 | -.01 | -.11 |
| Surg+Chemo | -.84 | .97 | -.08 | -.86 |  | -.84 | .84 | -.09 | -1.00 |
| Surg+Radio | 1.25 | 4.03 | .02 | .30 |  | .37 | 3.47 | .009 | .10 |
| Chemo+Radio | 1.64 | 2.29 | .07 | .71 |  | 1.37 | 1.97 | .06 | .69 |
| **Months from diagnosis** | -.005 | .01 | -.02 | -.31 |  | -.008 | .01 | -.05 | -.60 |
| **Employment** |  |  |  |  |  |  |  |  |  |
| Part-time | -2.02 | 2.27 | -.08 | -.89 |  | .66 | 1.95 | .03 | .33 |
| Seasional  Unemployment | 2.70  -1.23 | 4.02  1.75 | .06  -.09 | .67  -.70 |  | .61  1.41 | 3.45  1.50 | .01  .12 | .17  .93 |
| Self-employed | -6.19 | 3.26 | -.19 | -1.90 |  | -.51 | 2.80 | -.01 | -.18 |
| Sick leave | -1.96 | 1.49 | -.22 | -1.31 |  | 1.20 | 1.28 | .15 | .93 |
| Homemaker | -1.72 | 1.92 | -.10 | -.89 |  | 1.31 | 1.65 | .08 | .79 |
| Retirement | -1.00 | 1.61 | -.10 | -1.02 |  | 1.16 | 1.54 | .11 | .75 |
| **Cancer Type** |  |  |  |  |  |  |  |  |  |
| Lung | .84 | 2-15 | .03 | .39 |  | 4.57 | 1.85 | .22 | 2.47* |
| Colorectal | -4.43 | 1.44 | -.31 | -3.07** |  | -1.57 | 1.24 | -.12 | -1.27 |
| Gynecologic | -1.63 | 1.50 | -.09 | -1.08 |  | -2.17 | 1-29 | -.14 | -1.67 |
| **AAQ-II** | .24 | .04 | .54 | 6.07*** |  | .24 | .03 | .60 | 7.05*** |

Note. HADSA (Hospital Anxiety and Depression Scale-Anxiety) HADS (Hospital Anxiety and Depression Scale-Depression, AAQII (Action and Acceptance Questionnaire- II). Anxiety: *R^2^* = .22 for Step 1; ∆R^2^ = .23 for Step 2 (*p* < .001). Depression: *R^2^* = .22 for Step 1; ∆R^2^ = .28 for Step 2 (*p* < .001). * *p* < .05, ** *p* < .01, *** *p* < .001.

|  | *b* | SE *b* | *β* | *t* |
| --- | --- | --- | --- | --- |
| PTGI-SF  Model 1 |  |  |  |  |
| **Constant** | 52.58 | 5.97 |  | 8.80*** |
| **Age** | -.15 | .11 | -.13 | -1.39 |
| **Education** |  |  |  |  |
| Primary | 1.00 | 2.50 | .04 | .39 |
| Secondary | -1.63 | 2.56 | -.06 | -.64 |
| Advanced | 1.98 | 2.68 | .08 | .74 |
| **Months from Diagnosis** | .03 | .03 | .08 | .98 |
|  |  |  |  |  |
| Excluded Variables |  |  |  |  |
| **AAQ-II** | -.53 |  |  | -.59 |
